# Rifabutin boosts rifampicin accumulation in THP-1-derived M2 macrophages by inhibiting P-glycoprotein efflux activity

**DOI:** 10.1007/s00204-026-04350-x

**Published:** 2026-03-10

**Authors:** Katharina Hamburg, Cindy Bay, Jürgen Burhenne, Johanna Weiss, Julia C. Stingl, Dirk Theile

**Affiliations:** Internal Medicine IX - Department of Clinical Pharmacology and Pharmacoepidemiology, Medical Faculty Heidelberg, Heidelberg University Hospital, Heidelberg University, Im Neuenheimer Feld 410, 69120 Heidelberg, Germany

**Keywords:** THP-1, Macrophages, Polarization, P-glycoprotein, Rifampicin, Rifabutin

## Abstract

M2 macrophages show higher efflux activity of the P-glycoprotein (P-gp) drug transporter and lower rifampicin uptake than M1 macrophages. Accordingly, in M2 macrophages rifampicin accumulation should be restored by potent P-gp inhibitors such as rifabutin. THP-1 cells were differentiated (200 nM phorbol-12-myristate 13-acetate for 72 h) and polarized (20 ng/mL interleukin 4 and interleukin 13 for 48 h) to M2 macrophages. P-gp inhibition by rifabutin was assessed through flow cytometry using rhodamine 123 (substrate) and compared to zosuquidar (positive P-gp inhibitor control). Ultra-performance liquid chromatography coupled to mass spectrometry (UPLC-MS/MS) was used to evaluate the cellular rifampicin accumulation after 2 h of exposure to 0.05, 0.1, or 0.5 µM rifampicin, with or without co-treatment of cells with rifabutin (0.01 µM, 0.1 µM, 1 µM, 10 µM). Rifabutin (IC_50_ 0.8 µM) but not rifampicin inhibited P-gp efflux activity in M2 cells. When exposed to 0.05 µM rifampicin only, M2 cells accumulated 5.9 ± 1.1 ng rifampicin/ng protein, but took up significantly more rifampicin when co-treated with rifabutin (e.g. 10 µM rifabutin: 13.7 ± 3.5 ng rifampicin/ng protein). The same concentration-dependent boosting effect by rifabutin was detectable for the 0.1 µM and 0.5 µM rifampicin exposure levels. Together, rifabutin concentration-dependently inhibits P-gp and enhances rifampicin accumulation in M2 macrophages during concomitant drug exposure.

**Supplementary Information:**

The online version contains supplementary material available at 10.1007/s00204-026-04350-x.

## Introduction

During the chronic phase of tuberculosis (TB), immune cell-rich granulomas are formed. These multicellular conglomerates consist of T cells, natural killer cells (eliminating infected cells), B cells (producing antibodies), and polarized macrophages that phagocytose the tuberculosis-causing pathogen *mycobacterium tuberculosis* (Mtb) (Mattila et al. 2013). The balance of pro-inflammatory M1 macrophages and anti-inflammatory M2 macrophages in the granulomas is of special relevance. M1 macrophages exert antimicrobial effects by producing reactive oxygen species and nitric oxide and by maintaining the inflammatory anti-TB microenvironment. On the other hand, M2 macrophages limit the inflammatory response and secret various growth factors to promote fibrotic generation of granulomas (Thiriot et al. 2020). However, such M2-dominant granulomas are rather beneficial for the survival and proliferation of Mtb, given the provided immune tolerance or immune escape. Moreover, M2-enriched tuberculosis has been shown to associate with antibiotic drug resistance (Cho et al. 2020). From a pharmacological point of view, it seems reasonable to expect poor drug penetration into M2 cells, hindering the efficient eradication of the intracellular pathogen by antibiotics.

Drug transporters are well known to modulate the transport of therapeutic compounds across cell membranes, eventually affecting drug accumulation and therefore efficacy. Accordingly, previous investigations have evaluated if M2 cells exhibit a drug transporter profile that could explain poor antibiotic accumulation. Some experimental data indeed suggest that compared to M1 macrophages, M2 cells have higher expression and activity levels of the most important efflux transporter P-glycoprotein (P-gp, encoded by *ABCB1*) (Cory et al. 2016; Zha et al. 2013). However, there is less evidence whether the overexpression of drug transporters truly affects therapeutic drug accumulation or only the uptake of P-gp surrogate substrates such as fluorescent dyes that had been used in these investigations (Cory et al. 2016; Zha et al. 2013; He et al. 2018). In contrast, our recent work clearly demonstrated that (1) THP-1 cells-derived M2 macrophages show high *ABCB1* mRNA levels, (2) high functional P-gp efflux activity, and (3) THP-1 dell-derived M2 macrophages accumulate less rifampicin than M1 macrophages (Hamburg et al. 2026). Consequently, the inhibition of P-gp in M2 macrophages should prove the mechanistic role of P-gp and eventually restore rifampicin accumulation. In the present study, this approach was evaluated for the recently identified potent P-gp inhibitor rifabutin (Nilles et al. 2023; Theile et al. 2023; Phondeth et al. 2024).

## Materials & methods

### Materials

THP-1 cells were obtained from the American Type Culture Collection (ATCC, Manassas, USA) and RPMI 1640 medium was purchased from PanBiotech (Aidenbach, Germany). Penicillin–streptomycin, fetal calf serum (FCS), phorbol 12-myristate 13-acetate (PMA), interferon gamma (IFNγ), lipopolysaccharides (LPS) from *E. coli,* interleukin 4 and 13, rifampicin, rifabutin, rhodamine 123, and zosuquidar (LY335979) were purchased from Sigma-Aldrich (Taufkirchen, Germany). The Micro BCA™ Protein-Assay-Kit was bought from Thermo Fisher (Dreieich, Germany). [^2^H_8_]-Rifampicin was obtained from AppliChem (Darmstadt, Germany) and ^2^H_6_-rifabutin was purchased from AlsaChim (Strasbourg, France). The protease inhibitors pefabloc, leupeptin, pepstatin and aprotinin were purchased from Carl Roth (Karlsruhe, Germany). Ammonia (28%), acetonitrile (ACN), formic acid (HCOOH), and methanol (MeOH) were purchased from Merck (Darmstadt, Germany). Ultra-purified water was provided from the Arium® mini ultrapure water system (Sartorius, Göttingen, Germany).

### THP-1 cells and their differentiation and polarization

THP-1 cells were cultured in RPMI 1640 medium supplemented with 10% FCS and penicillin (100 U/mL)-streptomycin (0.1 mg/mL) at 5% CO_2_ and 37 °C. Differentiation and polarization were performed as described previously (Hamburg et al. 2026). Briefly, THP-1 monocytes were initially differentiated into M0 macrophages using 200 nM PMA for 72 h. After a recovery phase of five days, M0 macrophages were polarized for 48 h to anti-inflammatory M2 cells by treating them with 20 ng/mL interleukin 4 and 13 each.

### Assessment of P-gp inhibition by rifabutin

For the evaluation of P-gp inhibition in M2 cells, the rhodamine 123 efflux assay was used as described previously (Hamburg et al. 2026). Briefly, M2 cells were treated with 0.3 µM of the fluorescent P-gp substrate rhodamine 123 for 30 min at 37 °C under continuous shaking. During this time, rhodamine 123 is taken up into cells. After this loading period, cells were washed with 4 °C cold PBS and allowed to efflux rhodamine 123 during the following 30 min at 37 °C under continuous shaking. Rhodamine 123 efflux was either left unmodified (control) or modulated by concurrent co-treatment with 10 µM rifampicin, 10 µM rifabutin, or 10 µM of the specific P-gp inhibitor zosuquidar. Following this efflux phase, cells were washed two times and median fluorescence was recorded using a flow cytometer (MACSQuant analyzer 10, Miltenyi Biotec, Bergisch Gladbach, Germany). A total of 10,000 cells were used for analysis. The fluorescence ratio of drug-treated cells to non-treated cells was calculated.

Moreover, the concentration-dependency of P-gp inhibition was assessed for rifampicin (1–100 µM) and rifabutin (0.005–10 µM). The used concentration ranges were chosen based on cytotoxicity and previously observed P-gp inhibition potencies recorded in other cellular models (Nilles et al. 2023; Theile et al. 2023; Phondeth et al. 2024).

### Quantification of rifampicin uptake into M2 macrophages with or without rifabutin co-treatment

We measured intracellular rifampicin and rifabutin concentrations in M2 macrophages, which were treated with four different conditions. Treatment with rifampicin (0.05 µM, 0.1 µM and 0.5 µM) alone served as controls. These concentrations had been established to be non-toxic, to discriminate uptake characteristics in M1 and M2 cells, and to be clinically relevant (Hamburg et al. 2026). Additionally, the test groups were treated with these three rifampicin concentrations in combination with either 0.01 µM, 0.1 µM, 1 µM, or 10 µM of rifabutin, concentrations known to be likewise non-toxic and to be in range of expected P-gp inhibition potency (Nilles et al. 2023; Theile et al. 2023; Phondeth et al. 2024). Cells were incubated for 2 h, which was already proven to be sufficient to reach intracellular steady-state of rifampicin in M2 macrophages (Hamburg et al. 2026).

For the analysis of cellular rifampicin uptake into M2 macrophages, the published UPLC-MS/MS multi-method from our working group (Nilles et al. 2023) was adapted to a 96-well plate format. Also, we made alterations for measurement in THP-1-derived macrophages, which were validated as previously published (Hamburg et al. 2026). This multi-method allowed us to detect and quantify rifampicin and rifabutin in M2 macrophages simultaneously but separately (see Supplemental S1). Thus, this approach ensures that rifabutin is not incorrectly recorded as rifampicin during the co-treatment experiments. The analytic system consisted of a triple stage quadrupole mass spectrometer and an Acquity Classic UPLC® (Waters Xevo TQ-S, Milford, MA, USA). Mass spectrometric analysis was performed by multiple reaction monitoring using positive electrospray ionization.

Briefly, for cell lysis of M2 macrophages, acetonitrile/water (50/50, v/v) with 5% ammonium hydroxide was used. Protein precipitation was performed using acetonitrile. For internal standard, a final concentration of 600 ng/mL ^2^H_8_-rifampicin in combination with 30 ng/mL of ^2^H_6_-rifabutin was used.

Then, the plate was centrifuged for 10 min at 1000 g and supernatant was transferred into a 96-well plate, where the supernatant was evaporated with N_2_ and the residue was resuspended with water/acetonitrile (50/50, v/v) + 0.1% FA.

As described previously, protein samples for each control and test group and time point were harvested using lysis buffer, consisting of RIPA buffer and protease inhibitors. The protein concentration of each sample was determined by following the manufacturer’s instructions for the Micro BCA™ Protein-Assay-Kit. To calculate intracellular rifampicin concentrations, the measured [ng/mL] were normalized to protein concentration [ng/mL] and resulted in [ng_Rifampicin_/ng_Protein_] (Hamburg et al. 2026).

### Statistical analysis

The statistical analysis was performed using the GraphPad Prism 9.00 software (GraphPad Software, San Diego, USA). In order to analyse the data relating to P-gp inhibition and rifampicin uptake enhancement, an analysis of variance (ANOVA) with a Kruskal–Wallis test was employed.

A *P* value ≤ 0.05 was considered significant.

## Results

### P-gp inhibition in M2 cells by rifabutin

**Fig. 1:**
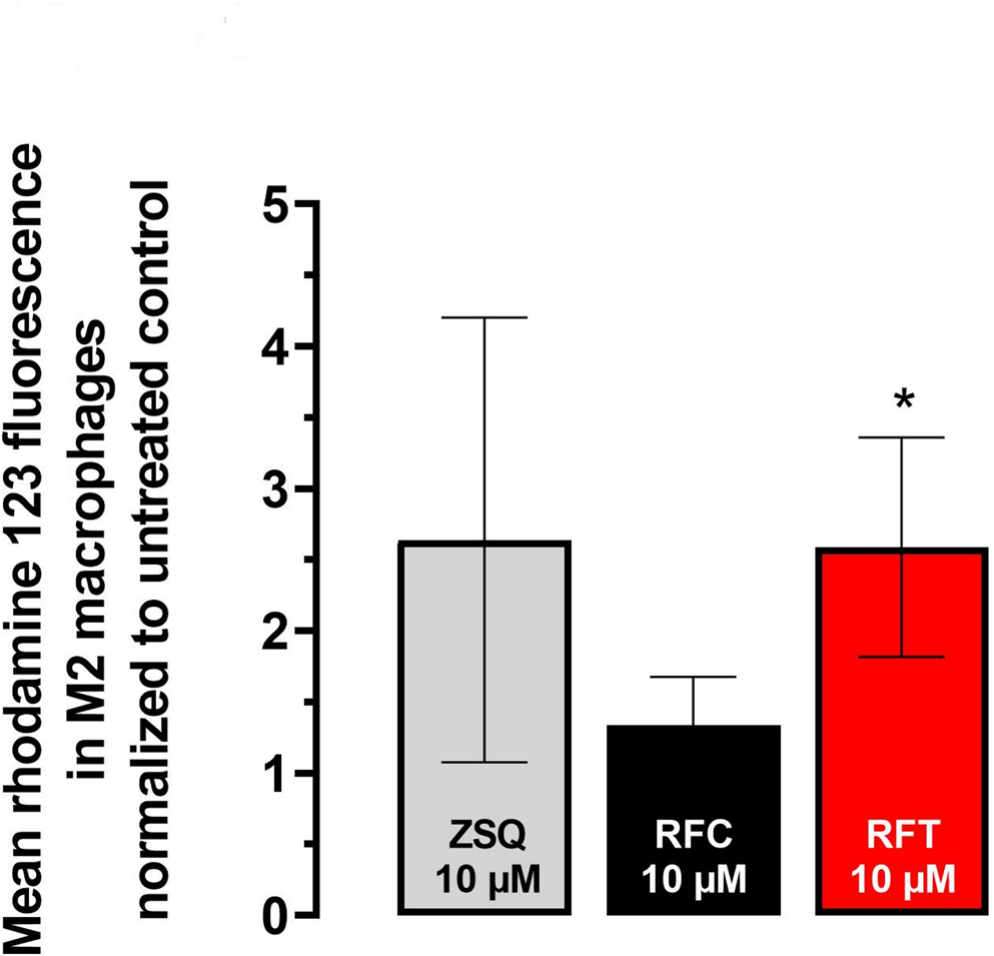
Impact of 10 µM of rifampicin (RFC, black), rifabutin (RFT, red), or zosuquidar (ZSQ, grey, positive control) on the rhodamine 123 fluorescence in M2 macrophages. Data are shown as mean ± S.D. of five independent experiments. Statistical significance between RFC and RFT treated cells was determined by ANOVA with Kruskal–Wallis and Dunn’s post-hoc test, controlling for multiple comparisons. A *P* value ≤ 0.05 was considered significant. *P < 0.05

When comparing mean rhodamine 123 fluorescence in drug-treated M2 macrophages to untreated controls, 10 µM rifampicin only had minor effects on mean rhodamine 123 fluorescence in M2 macrophages (1.3 ± 0.4-fold). In contrast, 10 µM rifabutin (2.6 ± 0.8-fold) increased rhodamine 123 fluorescence significantly more pronounced than rifampicin (*P* = 0.048). The positive control 10 µM zosuquidar (2.6 ± 1.6-fold) had comparable effects to rifabutin, suggesting similar P-gp inhibitory efficacy (Fig. 1).

**Fig. 2:**
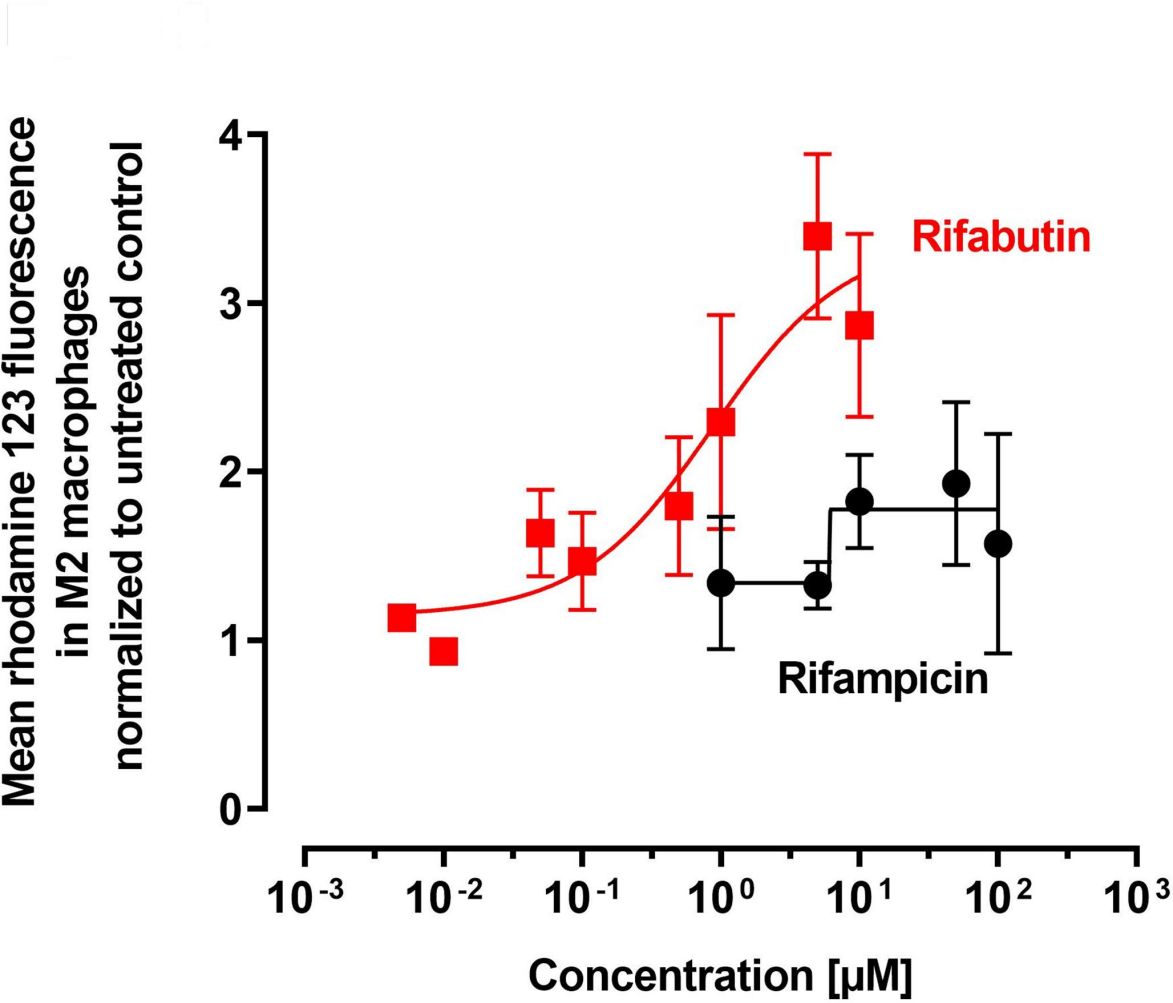
Concentration-dependent effect of rifampicin (black) or rifabutin (red) on the rhodamine 123 fluorescence in M2 macrophages. Data are shown as mean ± S.D. of three to four independent experiments in the rifabutin and four in the rifampicin group. Data of the log-transformed concentrations was fitted to a sigmoidal four-parameter fit

After this initial observation, we closely examined the potential concentration dependence of P-gp inhibition by rifabutin and rifampicin (Fig. 2). Again, treatment with rifampicin only had minor effects. Starting at 1.4 ± 0.4-fold (treatment with 1 µM rifampicin), rhodamine 123 fluorescence only reached 1.9 ± 0.5-fold (at 50 µM rifampicin) enhancement compared to untreated M2 cells, rendering the computation of a robust IC_50_ value impossible. In contrast, rifabutin concentration-dependently enhanced rhodamine 123 fluorescence in M2 macrophages from 1.1 ± 0.04-fold (when treated with 0.005 µM) to 3.4 ± 0.5-fold (when treated with 5 µM), resulting in a calculated IC_50_ of 0.8 µM, clearly suggesting potent inhibition of P-gp by rifabutin.

### Enhancement of rifampicin accumulation by co-treatment with rifabutin

**Fig. 3:**
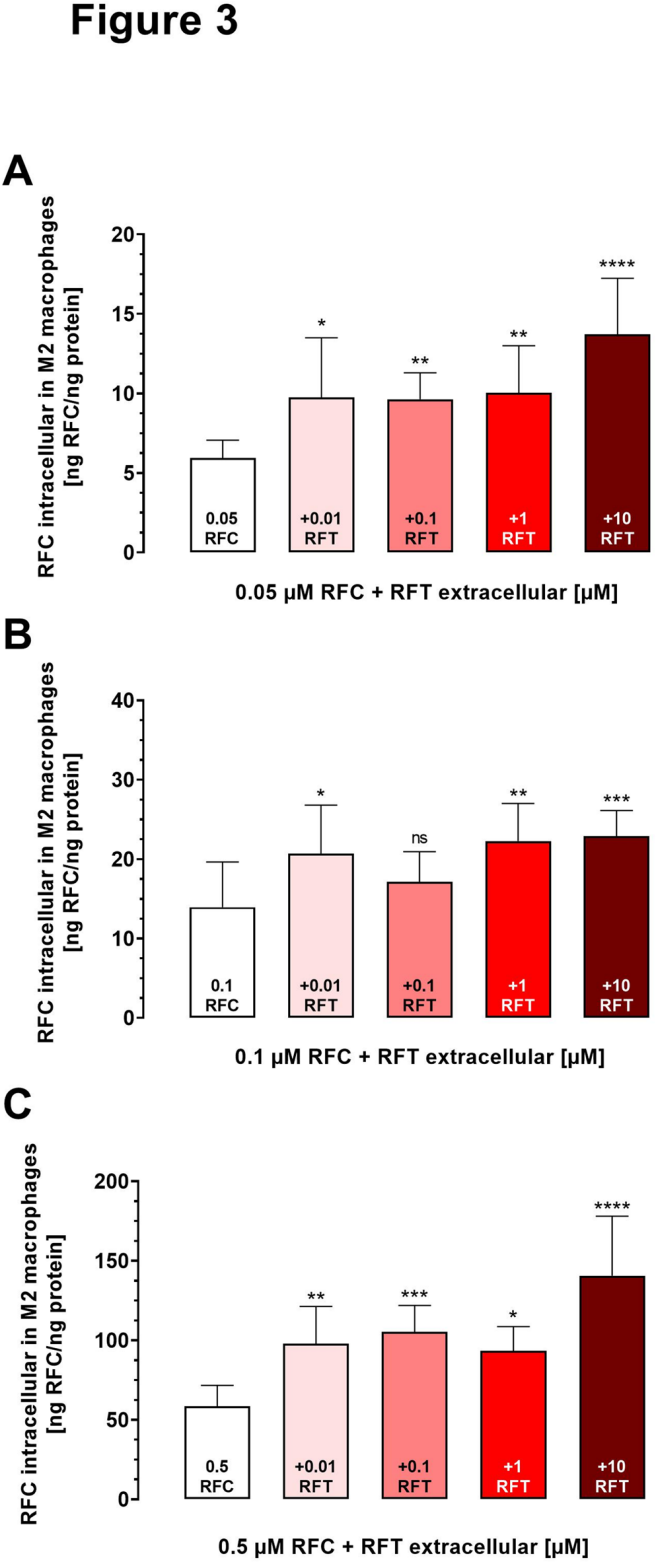
Impact of rifabutin (RFT, 0.01–10 µM) co-treatment on rifampicin (RFC) accumulation in M2 macrophages after 2 h exposure to 0.05 µM (**A**), 0.1 µM (**B**), or 0.5 µM RFC (**C**). Data are shown as mean ± S.D. of five independent experiments with technical triplicates each. Statistical significance was determined by ANOVA with Kruskal–Wallis test in which intracellular RFC concentrations without RFT were used as control. A *P* value ≤ 0.05 was considered significant. *P < 0.05; **P < 0.01; ***P < 0.001; **** *P* < 0.0001

To assess if rifabutin-mediated P-gp inhibition affects actual cellular accumulation of the P-gp substrate rifampicin, rifampicin uptake into M2 cells with or without concurrent rifabutin co-exposure was recorded via an UPLC-MS/MS method. Exposure to 0.05 µM rifampicin (Fig. 3A) resulted in an accumulation of 5.9 ± 1.1 ng rifampicin/ng protein. Co-treatment with only 0.01 µM rifabutin enhanced rifampicin uptake significantly to 9.7 ± 3.8 ng rifampicin/ng protein (*P* = 0.01). Co-treatment with 0.1 µM (9.6 ± 1.7 ng rifampicin/ng protein; *P* = 0.003) or 1 µM (10 ± 3 ng rifampicin/ng protein; *P* = 0.001) of rifabutin enhanced rifampicin uptake furthermore. Finally, at the highest co-treatment with 10 µM rifabutin, the intracellular rifampicin concentration was more than doubled to 13.7 ± 3.5 ng rifampicin/ng protein, compared to treatment with 0.05 µM rifampicin alone (*P* < 0.0001) (Fig. 3A).

These findings were also observed for the rifampicin concentrations of 0.1 µM (Fig. 3B) or 0.5 µM (Fig. 3C). In detail, extracellular rifampicin of 0.1 µM resulted in 14 ± 5.7 ng rifampicin/ng protein. Again, 0.01 µM rifabutin was sufficient to significantly enhance rifampicin uptake to 20.7 ± 6.1 ng rifampicin/ng protein (*P* = 0.03). At the highest rifabutin co-treatment concentration (10 µM), intracellular rifampicin was enhanced 1.5-fold to 22.9 ± 3.2 ng rifampicin/ng protein (*P* = 0.0001) (Fig. 3B). Treating M2 macrophages with 0.5 µM rifampicin alone resulted in intracellular concentrations of 58.5 ± 13.2 ng rifampicin/ng protein, which was enhanced almost two-fold by 0.01 µM of rifabutin (98 ± 23.3 ng rifampicin/ng protein; *P* = 0.006). Again, this trend continued and eventually led to a more than twofold increase of intracellular rifampicin levels to 140.6 ± 37.5 ng rifampicin/ng protein with 10 µM rifabutin co-treatment (*P* < 0.0001) (Fig. 3C).

## Discussion

The most important finding of this study is that rifabutin can enhance the cellular accumulation of rifampicin. For instance, exposure of M2 macrophages to 0.05 µM rifampicin led to intracellular levels of about 6 ng rifampicin/ng protein (Fig. 3A). However, when combined with 0.01 to 10 µM rifabutin, intracellular rifampicin levels increased significantly (e.g. 10 µM rifabutin boosting rifampicin accumulation 2.3-fold), depending on the rifabutin co-treatment concentrations. The same concentration-dependent rifabutin effect was observed for the 0.1 µM rifampicin exposure level (Fig. 3B) and the 0.5 µM rifampicin exposure level (Fig. 3C). At this point, it is crucial to discuss the used drug concentrations from a clinical point of view. For instance, if rifampicin distribution into the epithelial lining fluid of the lung is 30% (Clewe et al. 2015; Ziglam et al. 2002) compared to the plasma steady-state trough levels after administration of standard doses (C_min_: 0.006 µM (0.005 µg/ml), Chirehwa et al. 2015), rifampicin concentrations at the site of action are expected to be in the range of the concentrations used in our experiments. For rifabutin, plasma steady-state C_min_ was shown to be 0.084 µM (71.2 ng/mL; Ghannad et al. Plos One 2019) or 0.093 µM (78.9 ng/mL; Vourvahis et al. Antimicrob Agents Chemother 2012). Admittedly, there is no data on the distribution of rifabutin into the epithelial lining fluid of the lung, so definite data on the actual rifabutin concentrations at the site of action remain unknown. However, given its superior lipophilicity, its high volume of distribution (> 9 L/kg), and its known excellent cell and tissue penetration characteristics (Brogden et al. 1994; Skinner et al. 1995), one can expect that rifabutin reaches the lung at least as good as rifampicin. Together, in vivo exposure of alveolar M2 macrophages to 0.05 µM rifampicin and 0.01–0.1 µM rifabutin is very likely.

Besides the description of the favorable rifampicin-rifabutin interaction with clinically achievable concentrations, our data suggests a specific mode of boosting action of rifabutin. Using flow cytometry for the evaluation of rhodamine 123 accumulation (a fluorescent P-gp surrogate substrate), the data clearly demonstrated that rifabutin (but not rifampicin) is an effective inhibitor of P-gp on M2 macrophages. This finding perfectly agrees with our previous observations. In a series of in vitro experiments, the efficacy of rifabutin as a P-gp inhibitor was demonstrated in a variety of cell lines and models. These included human epithelial cells with genetically engineered human P-gp overexpressing (Theile et al. 2023), murine leukemia cells with murine *mdr1a/b* overexpressing (Nilles et al. 2023), LS180 cells with rifampicin-mediated human P-gp overexpressing (Phondeth et al. 2024), and a physiology-based pharmacokinetic modelling (Theile et al. 2023).

Moreover, an in silico molecular docking analysis identified the specific amino acids within the P-gp protein (e.g. glutamate 874) that the spiro piperidine group of rifabutin strongly interacts with (ΔG–11.5 kcal/mol), while typical substrates (e.g. paclitaxel) or non-inhibitors (e.g. rifampicin) do hardly interact with this inhibitor binding site (Phondeth et al. 2024). Together, this data proposes that rifabutin boosts rifampicin uptake into M2 macrophages by profound inhibition of P-gp.

This study has its limitations. First, we only examined M2 macrophages derived from the THP-1 cell line, which in fact is an established model for macrophage differentiation and polarization (Chanput et al. 2014), but certainly does not completely reflect the in vivo situation. Secondly, we only examined M2 macrophages and did not show whether the rifabutin booster effect can also be observed in M1 macrophages. However, M1 macrophages have a significantly lower P-gp efflux activity compared to M2 cells (Hamburg et al. 2026). Accordingly, inhibiting the poorly active P-gp in these M1 cells is not expected to lead to a substantial improvement of drug uptake. Third, non-polarized M0 macrophages were not evaluated as well, although previous work with THP-1 derived M0 macrophages demonstrated enhanced uptake of ethambutol and rifampicin during co-treatment with P-gp inhibitors like tariquidar (Hartkoorn et al. 2007). Also, there is data regarding enhanced uptake of berberine in RAW264.7 macrophages when treated with ritonavir and lopinavir (Zha et al. 2013). In consequence, this is the first study on rifampicin uptake into fully polarized M2 macrophages, being considerably affected by co-treatment with rifabutin. Also another strength of our study is that with our validated UPLC-MS/MS method, we are able to measure rifampicin and rifabutin separately, therefore, there is no interference of rifabutin in the intracellular measurement of rifampicin.

After discussing the data obtained and the respective weaknesses and strengths of our investigation, the question of how to proceed is imperative. Notwithstanding the findings of the increased rifampicin uptake into M2 macrophages, the question of whether this twofold concentration enhancement would actually lead to an improved rifampicin efficacy remains unresolved. For instance, there is a need for further research to determine whether there is a decrease in Mtb proliferation in M2 macrophages. Consequently, a series of subsequent experiments are recommended: Firstly, the treatment of Mtb-infected macrophages should be undertaken with mono-rifampicin or a rifampicin/rifabutin combination (at the same total drug concentration as mono-rifampicin). A comparison of the respective Mtb growth rates will provide insight into the significance of the drug combination from a microbiological perspective. Secondly, a physiology-based pharmacokinetic model comprising the lung and macrophages as distinct compartments can estimate the effects of full-blown P-gp inhibition on rifampicin’s distribution pattern. Finally, it is proposed that murine models of tuberculosis could be utilized to evaluate the pharmacokinetic and pharmacodynamic interaction of rifampicin and rifabutin in the future. Together, these experiments will demonstrate ‘the potential of P-gp inhibitors as an adjunct therapy’ in the treatment of tuberculosis (Parida et al. 2024).

## Conclusion

This is the first study demonstrating concentration-dependent inhibition of P-gp by rifabutin in THP-1-derived M2 macrophages, leading to enhanced uptake of rifampicin. The data additionally hints that low booster doses of rifabutin might be sufficient to significantly enhance cellular rifampicin accumulation. This finding thus provides a solution to impaired rifampicin uptake into M2 macrophages, a potential mode of rifampicin resistance in tuberculosis.

## Supplementary Information

Below is the link to the electronic supplementary material.

Supplementary Material 1

## Abbreviation

P-gp: P-glycoprotein
