# Supplementary material for: Rifabutin boosts rifampicin accumulation in THP-1-derived M2 macrophages by inhibiting P-glycoprotein efflux activity
Source: Arch Toxicol. 2026 Mar 10;100(6):2535–41. doi: 10.1007/s00204-026-04350-x (PMC13221408; doi:10.1007/s00204-026-04350-x)
Supplement: Supplementary file 1 — Supplementary Material 1 [file 204_2026_4350_MOESM1_ESM.docx]

**Supplementary material**

**
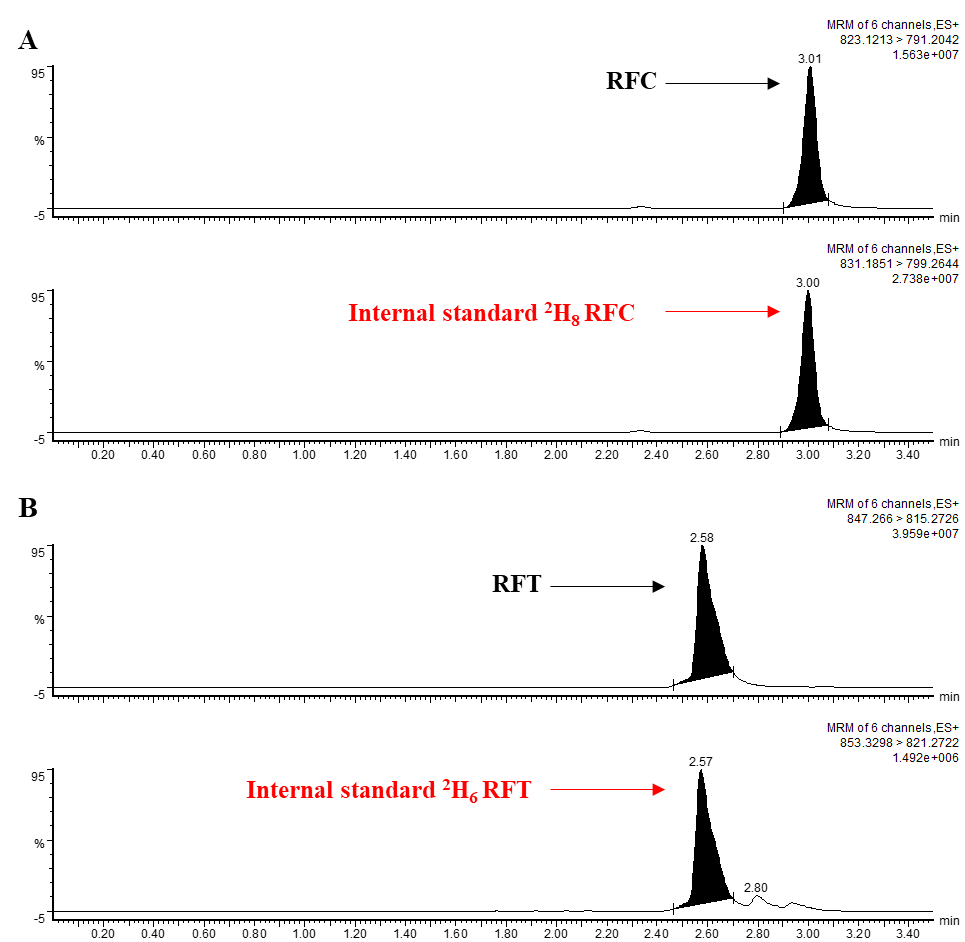
**

**Figure S1: UPLC-MS/MS -Chromatography of Quality Control sample C.**

Resulting chromatography peaks of quality control sample C (75 ng/mL) for rifampicin (RFC, A) and rifabutin (RFT, B). The graph shows the signal intensity [%] over time [min]. Internal standards of each compound are represented beneath. The intensity of signals is displayed in the upper right corner. The resulting retention times were 3.00 min for RFC and 2.58 min for RFT. The measurement consisted of a triple stage quadrupole mass spectrometer and an Acquity Classic UPLC®. The mass spectrometric analysis was performed by selective reaction monitoring using positive electrospray ionization.
